# Exploring Peracetic Acid and Acidic pH Tolerance of Antibiotic-Resistant Non-Typhoidal *Salmonella* and *Enterococcus faecium* from Diverse Epidemiological and Genetic Backgrounds

**DOI:** 10.3390/microorganisms11092330

**Published:** 2023-09-16

**Authors:** Andreia Rebelo, Bárbara Duarte, Ana R. Freitas, Luísa Peixe, Patrícia Antunes, Carla Novais

**Affiliations:** 1 UCIBIO-Applied Molecular Biosciences Unit, Laboratory of Microbiology, Department of Biological Sciences, Faculty of Pharmacy, University of Porto, 4050-313 Porto, Portugal; acr@ess.ipp.pt (A.R.); bduarte@ff.up.pt (B.D.); ana.freitas@iucs.cespu.pt (A.R.F.); lpeixe@ff.up.pt (L.P.); 2 Associate Laboratory i4HB—Institute for Health and Bioeconomy, Faculty of Pharmacy, University of Porto, 4050-313 Porto, Portugal; 3 School of Medicine and Biomedical Sciences (ICBAS), University of Porto, 4050-313 Porto, Portugal; 4 ESS, Polytechnic of Porto, 4200-072 Porto, Portugal; 5 1H-TOXRUN, One Health Toxicology Research Unit, University Institute of Health Sciences, CESPU, CRL, 4585-116 Gandra, Portugal; 6 Faculty of Nutrition and Food Sciences, University of Porto, 4150-180 Porto, Portugal

**Keywords:** biocides, one health, food chain, antimicrobial resistance

## Abstract

Acid stress poses a common challenge for bacteria in diverse environments by the presence of inorganic (e.g., mammals’ stomach) or organic acids (e.g., feed additives; acid-based disinfectants). Limited knowledge exists regarding acid-tolerant strains of specific serotypes, clonal lineages, or sources in human/animal pathogens: namely, non-typhoidal *Salmonella enterica* (NTS) and *Enterococcus faecium* (Efm). This study evaluated the acidic pH (Mueller–Hinton acidified with HCl) and peracetic acid (PAA) susceptibility of Efm (*n* = 72) and NTS (*n* = 60) from diverse epidemiological/genetic backgrounds and with multiple antibiotic resistance profiles. Efm minimum growth/survival pH was 4.5–5.0/3.0–4.0, and for NTS it was 4.0–4.5/3.5–4.0. Efm distribution among acidic pH values showed that only isolates of clade-non-A1 (non-hospital associated) or the food chain were more tolerant to acidic pH compared to clade-A1 (hospital-associated clones) or clinical isolates (*p* < 0.05). In the case of NTS, multidrug-resistant (MDR) isolates survived better in acidic pH (*p* < 0.05). The PAA MIC/MBC for Efm was 70–120/80–150 mg/L, and for NTS, it was 50–70/60–100 mg/L. The distribution of Efm among PAA concentrations showed that clade-A1 or MDR strains exhibited higher tolerance than clade-non-A1 or non-MDR ones (*p* < 0.05). NTS distribution also showed higher tolerance to PAA among non-MDR and clinical isolates than food chain ones (*p* < 0.05) but there were no differences among different serogroups. This unique study identifies specific NTS or Efm populations more tolerant to acidic pH or PAA, emphasizing the need for further research to tailor controlled measures of public health and food safety within a One Health framework.

## 1. Introduction

Acid stress poses a widespread challenge for bacteria in various natural and transient environments, where exposure to organic or inorganic acids is commonplace. It occurs through natural geochemical or microbial metabolic processes, in mammal and bird stomachs (pH 1.5–3.7), in specific cells during infection (e.g., macrophages phagolysosomes: pH 5.4–6.0), in acid-rich foods (e.g., citrus fruits), in acidified feed (pH 3.5–4.6), or through the exposure to acid-based disinfectants (pH < 5.0) [1,2,3,4]. Inorganic acids (e.g., HCl) occur in animal stomachs, while organic acids, such as formic, propionic or citric, are often used in animal feed as preservatives or to promote animal health and growth by modulating gut microbiota [5,6,7]. On the other hand, the peracetic acid (PAA) is an organic acid-based broad-spectrum biocide used for multiple purposes (applications or concentration allowed differ according to geographic regions) as hand disinfection (150–2000 mg/L) [8], disinfection of fresh produce (<80 mg/L, USA) [9], poultry carcass (<2000 mg/L, USA) [10], animal drinking water (25 mg/L), food processing equipment and food contact surfaces (20–3000 mg/L), animal feet/animal houses (100–5000 mg/L), clinical settings surfaces (125–1500 mg/L) or reduction in fecal bacteria counts in waste water/sewage before leaving treatment plants (1.5 mg/L in the effluent) [8]. PAA stands out among other biocides due to its notable advantages, including its rapid action and short contact time, effectiveness even in the presence of high organic loads, rapid biodegradability and minimal environmental impact (PAA breaks down into harmless by-products—acetic acid, oxygen, and water) [3,8,11]. The effectiveness of organic acids relies on their ability to penetrate cell membranes as protonated acids, allowing the undissociated forms to freely diffuse through the cell membrane into the cytoplasm at low pH [1,12]. Inside the cell, acid dissociation occurs as a result of the elevated pH, leading to the release of charged anions and protons that accumulate in the cytoplasm, disrupting crucial enzymatic activity and exerting detrimental effects on protein and DNA/RNA synthesis as well as the proton motive force [13]. Consequently, the cell’s ability to restore its cytoplasmic alkalinity is compromised, profoundly impacting vital processes such as cell growth, metabolism, nutrient absorption, substrate degradation, and the synthesis of proteins and nucleic acids [13,14,15,16]. In addition to the acid action of PAA, this biocide also damages cellular components and genetic material through the generation of reactive oxygen species [17,18,19]. On the other hand, inorganic acids (e.g., HCl in the stomach) primarily act by reducing the cytoplasmic pH of bacteria [13].

Bacteria have developed multiple strategies to respond to acid stress, including the production of neutralizing products (e.g., NH_3_), ATP consumption for proton elimination (e.g., decarboxylation of amino acids), efflux of anions through membrane pumps (e.g., F_1_-F_0_-ATPase proton pump), or membrane modifications (e.g., fluidity, lipid composition) [1,13,20], while the tolerance mechanisms to PAA are not fully understood [3]. While acidic environments are prevalent and bacteria have well-documented adaptive strategies to cope with them [1,21], the precise impact of these factors on the selection of acid-tolerant and/or antibiotic-resistant strains, particularly those relevant to human health, remains poorly understood.

Limited knowledge exists regarding the occurrence of acid-tolerant strains in the food chain where acid stress is common, namely those associated with human and/or animal infections or used as hygiene/safety indicators of drinking water, food and food contact surfaces, as the case of non-typhoidal *Salmonella enterica* (NTS) and/or *Enterococcus faecium* (Efm) [22,23,24,25,26,27,28]. NTS or *Enterococcus* spp. have adaptive responses to acid tolerance [29,30,31], enabling their survival in diverse acidic environments across the food chain and beyond [32,33], but studies concerning PAA susceptibility are scarce and for NTS have been showing variable levels of tolerance [34,35,36]. Moreover, most of the studies on NTS and Efm often overlook the inclusion of isolates from diverse epidemiological and genetic backgrounds impairing the understanding of particular strains (e.g., serogroups/serotypes, clonal lineages, source-related or antibiotic resistance ones), which are better selected in specific acidic environments [28,34,37,38,39,40]. The aim of this study was to assess the susceptibility to acidic pH and PAA of a comprehensive collection of antibiotic-resistant NTS and Efm strains from diverse epidemiological and genetic backgrounds.

## 2. Materials and Methods

### 2.1. Epidemiological Background of Bacterial Isolates

A collection of 132 isolates including Efm (*n* = 72) and NTS (*n* = 60) obtained in previous surveillance studies [32,33,41,42] and representative of different geographical regions, sources, time spans and genomic backgrounds was analyzed. Efm were isolated between 1996 and 2018 in Portugal, Spain, Tunisia and Angola from human (*n* = 29; 23 clinical isolates, 6 from fecal colonization of healthy human or long-term facility care patients), food chain (*n* = 42; 28 from poultry skin, 8 from piggeries, 3 from supermarket trout, 2 from bovine meat; 1 from ready-to-eat salad) and environmental (*n* = 1) sources. In previous studies, these Efm were identified as clade A1 (*n* = 21; mostly associated in the literature with human infections and hospital outbreaks) or non-clade A1 (*n* = 37, non-hospital associated, mostly associated with animal colonization) [43], while some were not typed (*n* = 14). Sixty-one (85%) were multidrug-resistant (MDR; resistant to three or more antibiotics from different families) [44], with 25 showing resistance to vancomycin and 44 showing resistance to ampicillin. Only two isolates were susceptible to all antibiotics tested in previous studies. Efm data concerning acidic pH and PAA susceptibility from poultry were previously published [33] but included here for source comparison.

NTS were recovered between 2002 and 2018 in Portugal from human (*n* = 20 clinical isolates), food chain (n = 37; 12 from pork meat/pork products; 15 poultry meat/skin and by-products; 5 from pig and piggeries; 4 from trout aquaculture; 1 chicken manure, animal feed, surface/drain and foodstuff each) sources. They belong to 17 NTS serotypes (23 *S.* Typhimurium and its variant 1,4,[5],12:i:-, 4 *S.* Heidelberg, 4 *S.* Rissen, 4 *S.* Infantis, 3 *S.* Derby, 3 *S.* Enteritidis, 3 *S.* Mbandaka, 3 *S.* Virchow, 3 *S.* Stanley, 3 *S*. Hadar, 2 *S.* Kentucky, and 1 *S.* Bovismorbificans, *S.* Abony, *S.* Guerin, *S.* Linguere, *S.* Newport, each). Most were from serogroups B (*n* = 33) or C (*n* = 23). Forty-four were MDR, with 9 being resistant to ciprofloxacin or pefloxacin, 6 being resistant to colistin and 5 being resistant to cefotaxime. Eleven isolates did not show any resistance to the antibiotics tested in previous studies. NTS data concerning acidic pH and PAA susceptibility from 5 poultry isolates were previously published [24] but are included here for source comparison.

### 2.2. Susceptibility to Acidic pH

Susceptibility to acidic pH was performed using an adaptation of the microdilution standard method (ISO 20776-1:2019) [32,33,45]. To determine the minimum growth pH and survival pH of the bacteria, Mueller–Hinton II broth (BD BBL™, Franklin Lakes, NJ, USA) culture media was used and adjusted to a pH range of 2.0 to 6.5 (in 0.5 intervals) using hydrochloric acid (HCl) (Merck, Darmstadt, Germany). A freshly prepared 96-well microtiter plate was used for each assay. Bacterial suspensions in log-phase growth were adjusted and inoculated in each well with the corresponding pH to reach a final inoculum of 5 × 10^5^ CFU/mL. To confirm the inoculum for each isolate tested, colony counts were performed on the surface of Mueller–Hinton 2 agar plates (bioMérieux, Marcy-l’Étoile, France). The microdilution and the Mueller–Hinton 2 agar plates were incubated at 37 °C for 20 ± 2 h. The minimum growth pH was determined by identifying the lowest pH at which visible bacterial growth was observed. To determine the minimum survival pH, 10 μL of the wells without visible growth were inoculated on BHI agar (Liofilchem, Roseto degli Abruzzi, Italy) and incubated at 37 °C for 48 h. The minimum survival pH was determined as the lowest pH showing at least one colony growth in BHI agar. Control strains *Enterococcus faecalis* ATCC 29212 (minimum growth pH = 4.5; minimum survival pH = 4.0), *Escherichia coli* ATCC 25922 (minimum growth pH = 4.5; minimum survival pH = 3.5) and *Salmonella* Typhimurium LT2 (minimum growth pH = 4.0; minimum survival pH = 4.0) were included in all assays. All acidic pH susceptibility assays were performed in duplicate. The mean of the replicas was calculated as the final result for each isolate.

### 2.3. Susceptibility to PAA

Susceptibility to PAA was performed using an adaptation of the microdilution standard method (ISO 20776-1:2019) [45]. The Minimum Inhibitory Concentration (MIC_PAA_) was determined by identifying the first concentration of PAA without visible growth in Mueller–Hinton II broth supplemented with PAA (15% stock solution, CAS No. 79-21-0; PanReac AppliChem, Darmstadt, Germany) at concentrations ranging from 50 to 90 mg/L for NTS and 60 to 160 mg/L to Efm, both with a 10 mg/L interval, and distributed in a freshly prepared 96-well microtiter plate for each assay. Bacterial suspensions in log-phase growth were adjusted and inoculated in each well with the corresponding PAA concentration to reach a final inoculum of 5 × 10^5^ CFU/mL, confirmed for each isolate by colony counts in Mueller–Hinton 2 agar, which was followed by incubation at 37 °C for 20 ± 2 h. The Minimum Bactericidal Concentration (MBC_PAA_) was established as the lowest PAA concentration for which the number of colonies was equal to or less than the rejection value defined by CLSI:1999 (former NCCLS:1999) guidelines [46] based on the final bacterial inoculum of each well after incubation confirmed by actual count. To determine the MBC_PAA_, 10 μL of the wells without visible growth was plated on BHI agar (37 °C for 24–48 h). The pH of PAA concentrations tested was also determined for each assay, ranging between 5.5 and 7.0, in which non-dissociated PAA was present at 99.8–98% (PAA pKa = 8.2 at 20 °C) [47]. Control strains *E. faecalis* ATCC 29212 (MIC_PAA_ = 100 ppm; MBC_PAA_ = 120 ppm), *E. coli* ATCC 25922 (MIC_PAA_ = 60 ppm; MBC_PAA_ = 60 ppm) and *Salmonella* Typhimurium LT2 (MIC_PAA_ = 50 ppm; MBC_PAA_ = 70 ppm) were included in all assays. All PAA susceptibility assays were performed in duplicate. The mean of the replicas was calculated as the final result of each isolate.

### 2.4. Statistical Analysis

Differences in distribution of acidic pH and PAA susceptibility values among Efm or NTS isolates considering sources, clonal lineages, serogroups or susceptibility to antibiotics were analyzed by the Mann–Whitney test (α = 0.05), and comparison between proportions was measured by the Fisher exact test (α = 0.05), using Prism software, version 8.1.1 (GraphPad Software, Boston, MA, USA).

## 3. Results and Discussion

### 3.1. Susceptibility to Acidic pH

#### 3.1.1. *Enterococcus faecium*

**Figure 1:**
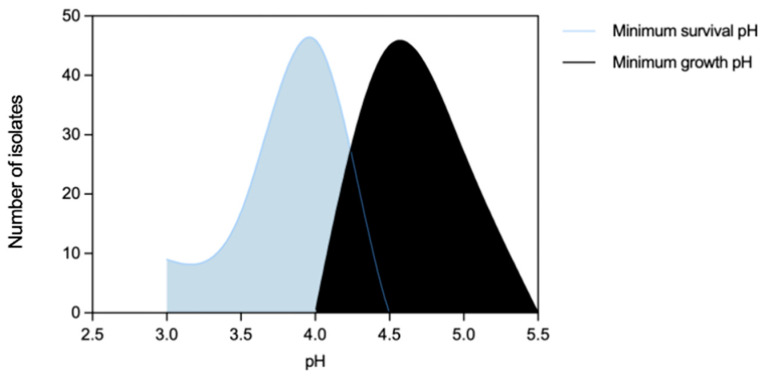
Distribution of *Enterococcus faecium* (*n* = 72 isolates) by acidic pH values corresponding to minimum survival pH and minimum growth pH.

**Figure 2:**
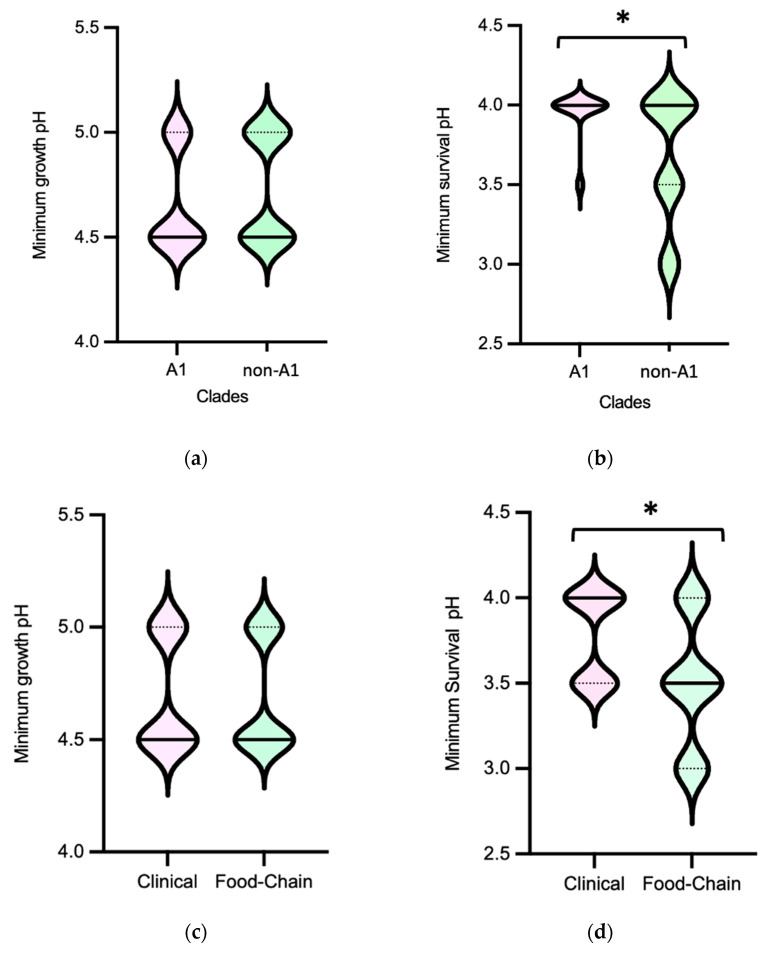

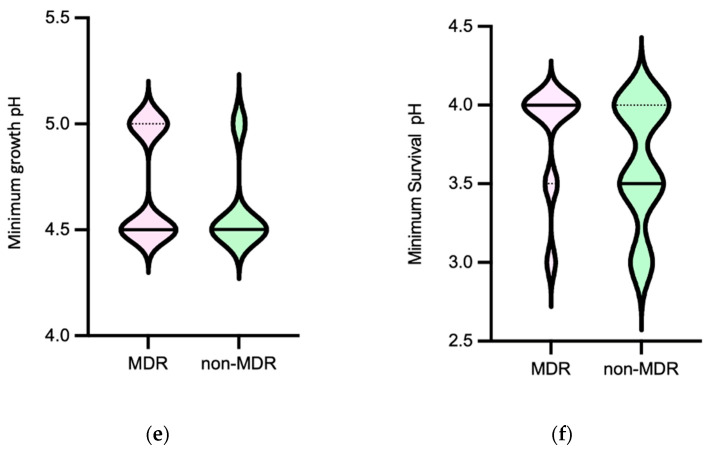
Distribution of *Enterococcus faecium* (*n* = 72 isolates) by minimum growth pH and minimum survival pH values according to clade type (**a**,**b**), source (**c**,**d**) and MDR profile (**e**,**f**). The central full line in the middle of each violin graph corresponds to the median value, and the dotted lines correspond to quartiles. The * represents a statistically significant result (*p* < 0.05, Mann–Whitney test). Abbreviations: MDR, multidrug resistant.

Efm exhibited minimum growth pH and minimum survival pH ranges of 4.5–5.0 and 3.0–4.0, respectively (Figure 1). In terms of minimum survival pH, the distribution of isolates from clade non-A1 (*n* = 37) suggests greater tolerance compared to those from clade A1 (*n* = 21) (*p* < 0.05) (Figure 2b). Also, the food chain isolates (*n* = 42) distribution suggests higher tolerance compared to the clinical isolates (*n* = 23) (*p* < 0.05) (Figure 2d). The comparison of Efm from environmental sources or human colonization was not considered due to the limited number of isolates available. In contrast, statistical analysis revealed no significant differences (*p* > 0.05) in the minimum survival pH of Efm among MDR and non-MDR isolates (Figure 2f). Regarding the minimum growth pH of isolates, identical results were observed comparing different clades, sources, or MDR/non-MDR profiles (*p* > 0.05) (Figure 2a,c,e). The 45 isolates with the lowest minimum growth at pH = 4.5 were from diverse sources (clinical, *n* = 13; human colonization, *n* = 5; food chain, *n* = 26; environment, *n* = 1) or clades (A1, *n* = 14; non-A1, *n* = 20; non-identified, *n* = 11), with most being MDR (*n* = 36). The nine isolates with the lowest value of minimum survival at pH = 3.0 were recovered from poultry meat (*n* = 7) raised in farms using organic acids in feed and recovered in a slaughterhouse using PAA as a sanitizer [33] as well as from supermarket trout (*n* = 2), most belonging to clade non-A1 (*n* = 7) and being MDR (*n* = 7). Efm resistant to clinically relevant antibiotics vancomycin or ampicillin showed similar minimum growth pH or minimum survival pH values to other isolates susceptible to these antibiotics.

The pH values at which Efm grew and survived in this study were consistent with previous findings for this species [37,39,48]. Isolates from diverse sources and antibiotic resistance profiles tolerate acidic pH values, supporting Efm widespread in diverse environments and hosts. However, the observation of greater tolerance in Efm isolates recovered from the food chain or belonging to clade non-A1 (usually identified in animal colonization), compared to clinical isolates or those from clade A1 (usually identified in human infection and hospital outbreaks), suggests distinct adaptive needs for acid stress in subpopulations that have adapted to different niches. The increased tolerance of Efm isolates from food–animal sources may be attributed to their exposure to acidic environments during food production, processing, and preservation [6,49,50]. In fact, the most recent poultry isolates included in the study were recovered from chicken meat, which had been sourced from chickens fed with diets supplemented with organic acids [33]. Furthermore, the absence of significant differences in minimum growth pH and minimum survival pH between MDR and non-MDR isolates suggests that acidic environments may not be a significant driver selecting such strains. However, it is worth noting that even at such low pH (3.0–3.5), the survival of antibiotic-resistant Efm strains, such as those resistant to clinically relevant vancomycin and ampicillin, is not impaired, as for example during passage through the stomach and gut of both humans and animals.

#### 3.1.2. Non-Typhoidal *Salmonella*

**Figure 3:**
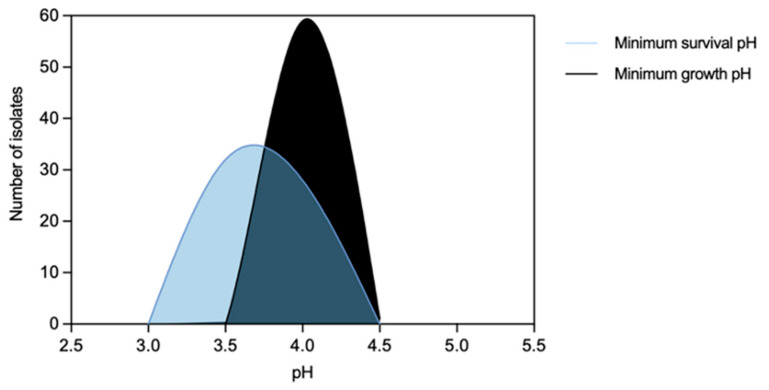
Distribution of non-typhoidal *Salmonella* (*n* = 60 isolates) by acidic pH values corresponding to minimum survival pH and minimum growth pH.

**Figure 4:**
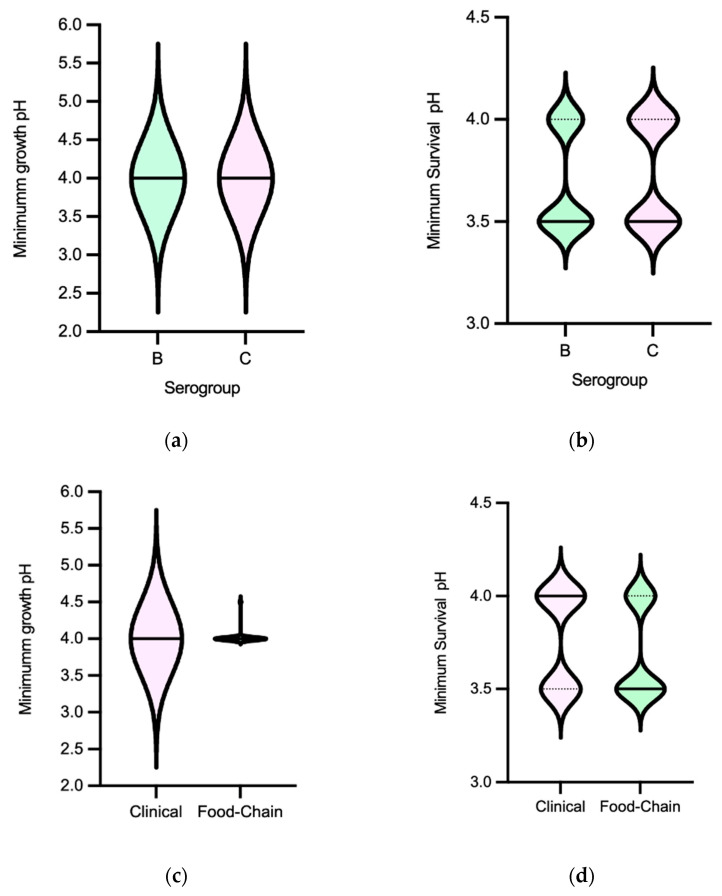

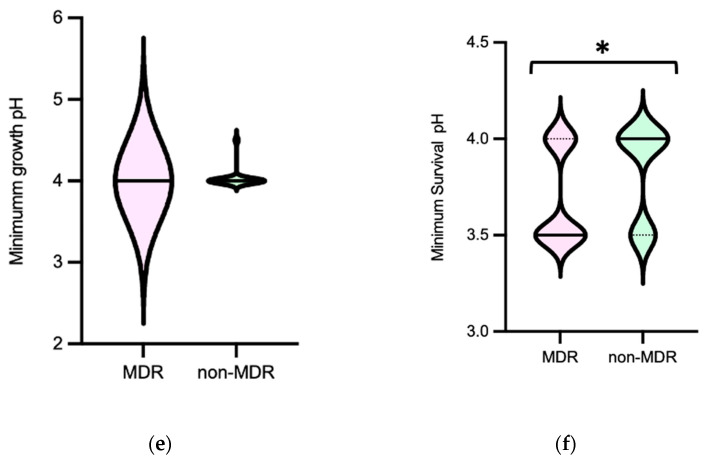
Distribution of non-typhoidal *Salmonella* (*n* = 60 isolates) by minimum growth pH and minimum survival pH values according to serogroup (**a**,**b**), source (**c**,**d**) and MDR profile (**e**,**f**). Central full line in the middle of each violin graph corresponds to the median value and the dotted lines correspond to quartiles. The * represents a statistically significant result (*p* < 0.05, Mann–Whitney test). Abbreviations: MDR, multidrug resistant.

Minimum growth pH was for most isolates 4.0, with just one *S.* Typhimurium from a pig carcass showing 4.5. The minimum survival pH for NTS ranged between 3.5 and 4.0, (Figure 3). MDR isolates distribution suggest they survive better in lower pH values than non-MDR ones (Figure 4f). No statistical differences were observed among minimum growth pH and minimum survival pH when comparing isolates from different sources with/without an MDR profile (except for minimum growth pH) or serogroups (B and C) (Figure 4a–d). The 32 isolates with the lowest value of minimum survival at pH = 3.5 were recovered from different samples of food chain (*n* = 23) and patients (*n* = 9) with most being from serogroup B (*n* = 20) and MDR (*n* = 26). NTS isolates resistant to the clinically relevant antibiotics ciprofloxacin or pefloxacin, 3rd generation cephalosporins or colistin showed minimum growth pH or minimum survival pH values similar to isolates susceptible to these antibiotics.

The pH tolerance values observed for NTS were consistent with those reported in the literature for the serotypes frequently associated with human infections, specifically *S.* Typhimurium (serogroup B) [51,52,53]. The lack of variation in acid tolerance from serogroups B (*n* = 33) and C (*n* = 23) or sources may be explained by the fact that NTS is a zoonotic pathogen, with most isolates of the serogroups studied being similarly adapted to the diverse food chain environment acid challenges. In contrast to Efm, there was an association between acid tolerance and MDR NTS, suggesting that acid stress may play a role in selecting such populations. It is also important to note that NTS ability to tolerate acidic conditions facilitates its survival and passage through the gastrointestinal tracts of humans and animals, including strains from this study showing resistance to clinically relevant antibiotics such as quinolones, 3rd generation cephalosporins, or colistin, especially of the emergent serotypes belonging to serogroups B or C often causing human infections [26,54].

### 3.2. Susceptibility to PAA

#### 3.2.1. *Enterococcus faecium*

**Figure 5:**
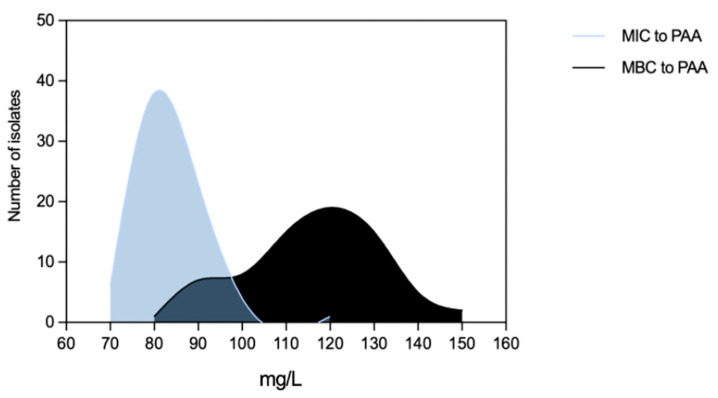
Distribution of *Enterococcus faecium* (*n* = 72 isolates) by Minimum Inhibitory Concentrations (MICs) and Minimum Bactericidal Concentrations (MBCs) of peracetic acid.

**Figure 6:**
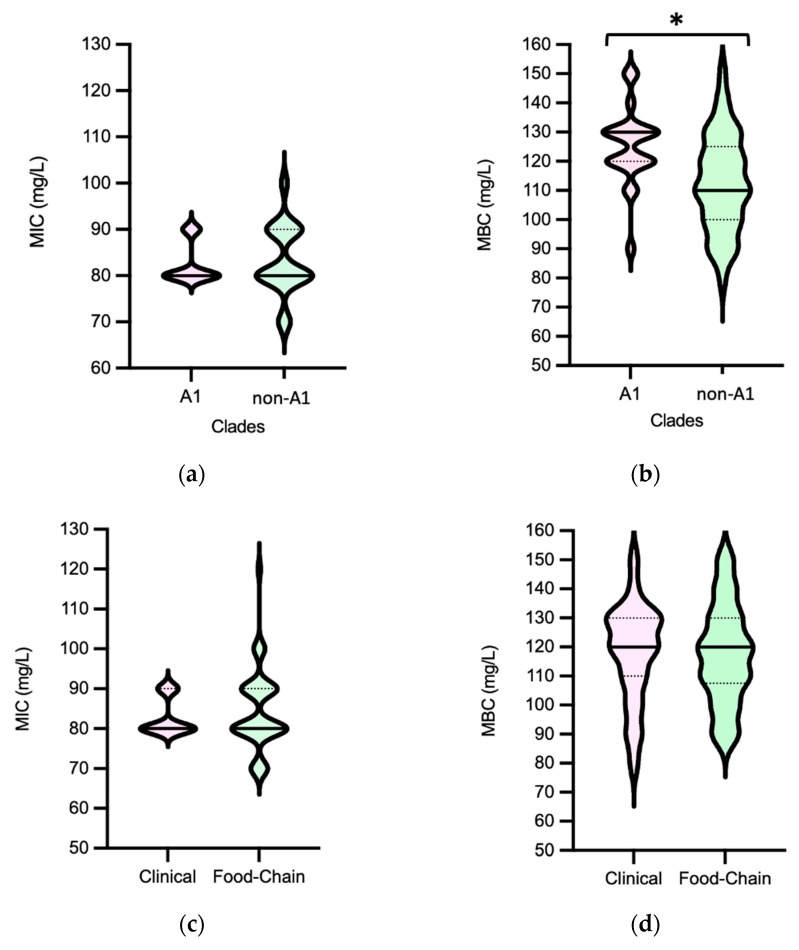

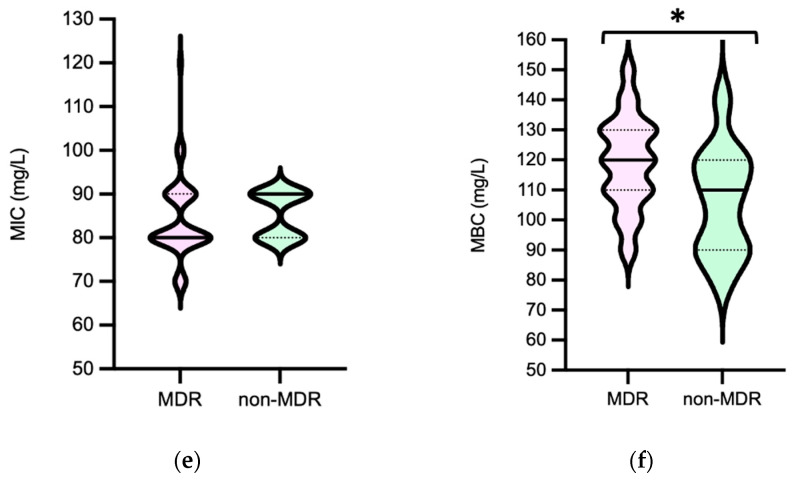
Distribution of *Enterococcus faecium* (*n* = 72 isolates) by Minimum Inhibitory Concentrations (MICs) and Minimum Bactericidal Concentrations (MBCs) of peracetic acid according to clade type (**a**,**b**), source (**c**,**d**) and MDR profile (**e**,**f**). Central full line in the middle of each violin graph corresponds to the median value and the dotted lines correspond to quartiles. The * represents a statistically significant result (*p* < 0.05, Mann–Whitney test). Abbreviations: MDR, multidrug resistant.

**Figure 7:**
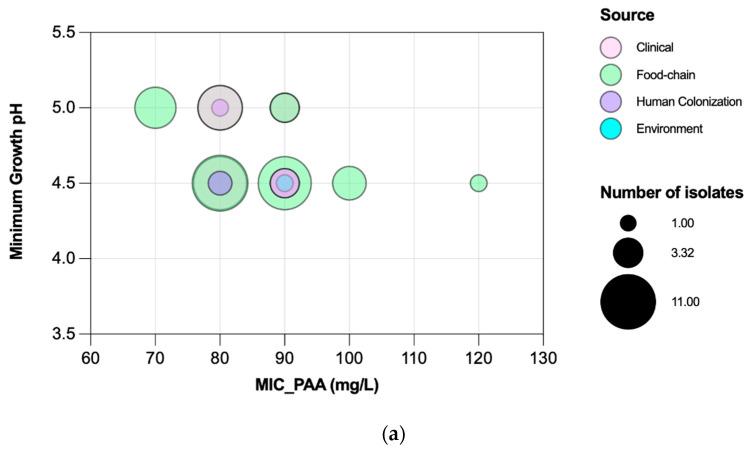

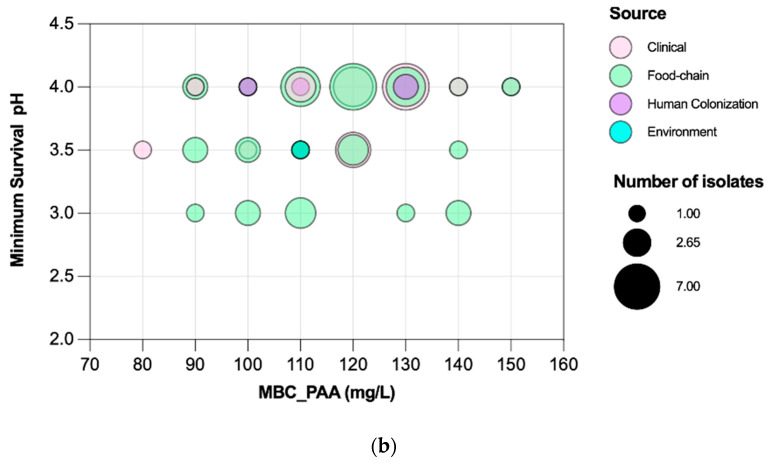
Association of peracetic acid Minimum Inhibitory Concentrations (MICs) with minimum growth pH values (**a**), and peracetic acid Minimum Bactericidal Concentrations (MBCs) with minimum survival pH values (**b**) of *Enterococcus faecium* (*n* = 72 isolates). The gray color means there is an overlapping of pink (clinical) and green (food-chain) isolates.

Efm MIC_PAA_ and MBC_PAA_ varied in the ranges of 70–120 mg/L and 80–150 mg/L, respectively (Figure 5). Efm from clade A1 (*n* = 21) or with an MDR profile were more tolerant to PAA considering MBC distribution values compared with those of clade non-A1 or non-MDR, respectively (*p* < 0.05) (Figure 6b,f). Most of the clade A1 isolates (52%, *n* = 11/21) had the highest values of MBC_PAA_ = 130–150 mg/L compared to clade non-A1 (24%, *n* = 9/37) (*p* < 0.05). The 20 isolates showing the higher values of MBC_PAA_ (130, 140 or 150 mg/L) were associated with the hospitalization of long-care facility patients (*n* = 10), with poultry meat collected in a slaughterhouse using PAA as a disinfectant (*n* = 6), and the remaining four from piggeries environment and healthy human feces. No statistical differences were observed when comparing MBC_PAA_ values of Efm from different sources (Figure 6d). Regarding MIC_PAA_, similar results were observed comparing clades, sources or antibiotic resistance profiles (Figure 6a,c,e). The five isolates with the highest MIC_PAA_ = 100–120 mg/L were all recovered from poultry meat from a slaughterhouse using PAA as a sanitizer and organic acids in feed [33], belonged to clade non-A1 (*n* = 2) or were non-identified (*n* = 2), and were MDR. Vancomycin or ampicillin-resistant Efm showed MIC_PAA_ = 80–90 mg/L or 70–90 mg/L, respectively, and MBC_PAA_ = 90–150 mg/L, each. Our analysis of Efm from all sources revealed no significant association between MIC_PAA_ and minimum growth pH or between MBC_PAA_ and minimum survival pH (*p* > 0.05) (Figure 7).

As far as we know, there are limited studies showing the susceptibility of Efm populations to PAA, and they use diverse methodological strategies [40,55,56], which makes it difficult to compare our data with isolates from other collections. However, this study shows that Efm can survive above the minimum concentration of PAA used in the food chain (20 mg/L sanitation of automatic spraying in closed systems; 125 mg/L hand disinfection) and in the clinical settings (125 mg/L hand disinfection in hospitals, health and animal care areas) [8]. Although similar proportions of clinical Efm presented MBC above or below the 125 mg/L (*n* = 9/23 isolates and *n* = 14/23, respectively; *p* > 0.05), most clade A1 isolates were above this value, suggesting they can at least survive hand disinfection. This concern extends to lower concentrations of PAA used in food industry surfaces or those expected to occur in sewage effluents (1.5 mg/L) [8], as they may not completely eliminate all Efm strains. This limitation in efficacy against such hygiene or fecal indicators raises concerns about the containment of antibiotic-resistant isolates in the environment. Thus, strains exhibiting higher MBCs warrant careful surveillance across different settings.

While a recent study indicated that exposure of Efm to low doses of PAA did not lead to changes in the relative abundance of the highly prevalent transferable *erm(B)* gene (macrolide, lincosamide, and streptogramin B resistance), despite bacterial adaptation to PAA stress [57], further data are needed to evaluate whether PAA is an effective choice as a sanitizer that does not promote the selection of antibiotic-resistant Efm. This evaluation becomes particularly important in the clinical setting where this species is a major pathogen.

#### 3.2.2. Non-Typhoidal *Salmonella*

**Figure 8:**
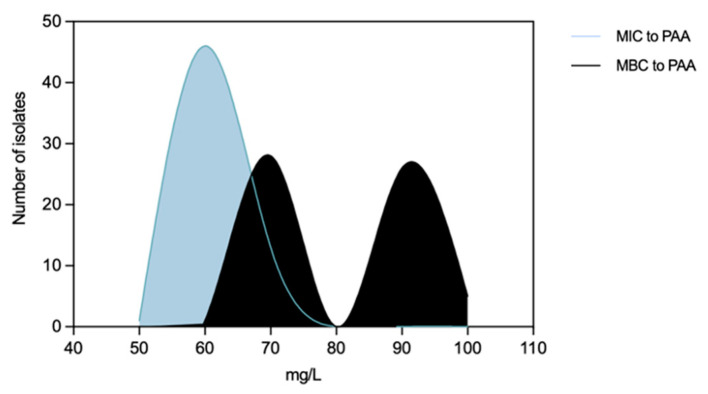
Distribution of non-typhoidal *Salmonella* (*n* = 60 isolates) by Minimum Inhibitory Concentrations (MICs) and Minimum Bactericidal Concentrations (MBCs) to peracetic acid.

**Figure 9:**
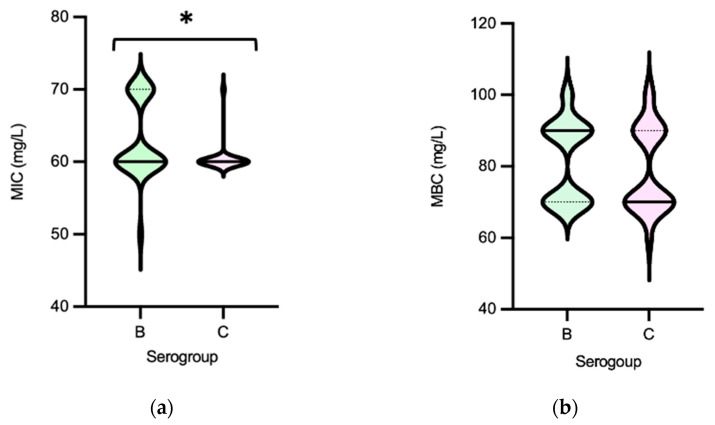

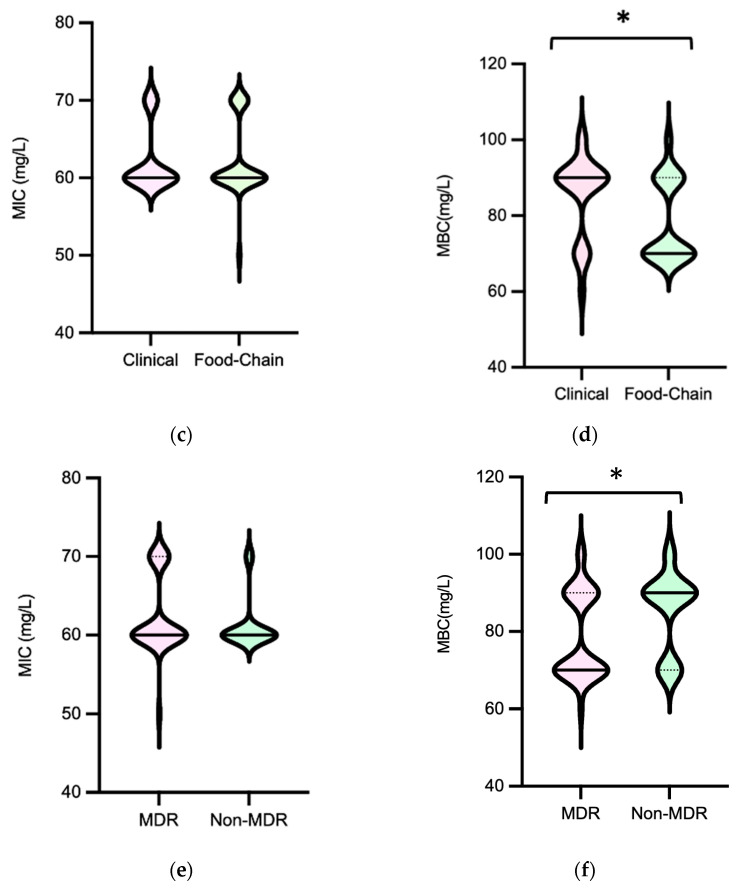
Distribution of non-typhoidal *Salmonella* (*n* = 60 isolates) by Minimum Inhibitory Concentrations (MICs) and Minimum Bactericidal Concentrations (MBCs) to peracetic acid according to serogroups (**a**,**b**), source (**c**,**d**) and MDR profile (**e**,**f**). Central full line in the middle of each violin graph corresponds to the median value and the dotted lines correspond to quartiles. The * represents a statistically significant result (*p* < 0.05, Mann–Whitney test). Abbreviations: MDR, multidrug resistant.

**Figure 10:**
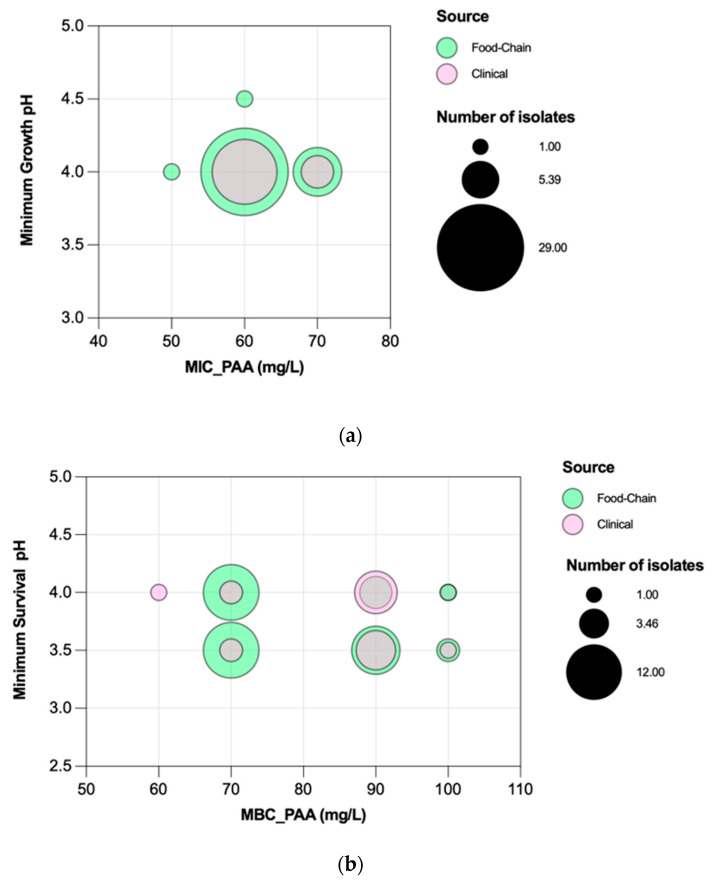
Association of peracetic acid Minimum Inhibitory Concentrations (MICs) with minimum growth pH values (**a**) and Peracetic Acid Minimum Bactericidal Concentrations (MBCs) with minimum survival pH values (**b**) of non-typhoidal *Salmonella* (*n* = 60 isolates). The gray color means there is an overlapping of pink (clinical) and green (food chain) isolates.

*Salmonella* MIC_PAA_ and MBC_PAA_ varied in the ranges of 50–70 mg/L and 60–100 mg/L, respectively (Figure 8). Isolates belonging to serogroup B demonstrated a greater ability to grow under PAA compared to those from serogroup C, as indicated by the MIC_PAA_ distribution (*p* < 0.05) (Figure 9a), but they were not able to survive better, as similar values of MBCs were found (*p* > 0.05) (Figure 9b). Moreover, both human-associated infections and non-MDR isolates exhibited higher tolerance to PAA as reflected by the MBC (Figure 9d,f). Of note, 75% of the isolates causing human infection (*n* = 15/20) or that were non-MDR (*n* = 12/16) showed the highest values observed (MBC_PAA_ = 90–100 mg/L; *p* < 0.05), contrasting with those of the food chain (40%, *n* = 16/40) or MDR (43%, *n* = 19/44), respectively. No statistical differences were observed when comparing the MIC_PAA_ of NTS from different sources or with MDR or non-MDR profiles (Figure 9c,e). The 13 isolates with the highest MIC_PAA_ = 70 mg/L were mostly from the food chain (*n* = 9) followed by patients (*n* = 4); most belonged to serogroups B (*n* = 11), C or D (one each), and 11 were MDR. The five isolates with the highest value of MBC_PAA_ = 100 mg/L were recovered from the food chain (*n* = 3) and patients (*n* = 2), belong to serogroups B (*n* = 3) or C (*n* = 2), and three were MDR. Most of the ciprofloxacin or pefloxacin-resistant isolates had an MIC_PAA_ = 60–70 mg/L and MBC_PAA_ = 70–100 mg/L, those resistant to colistin had an MIC_PAA_ = 50–70 mg/L and MBC_PAA_ = 90–100 mg/L, and those resistant to 3rd-generation cephalosporins had an MIC_PAA_ = 60–70 mg/L and MBC_PAA_ = 70 mg/L. No association between MIC_PAA_ and minimum growth pH or between MBC_PAA_ and minimum survival pH was detected (Figure 10) in the NTS from all sources analyzed (*p* > 0.05), as isolates with high or low pH growth or survival presented diverse MIC_PAA_ or MBC_PAA_ values.

Our findings emphasize that under certain conditions, the currently recommended concentrations of PAA used in disinfection products applied in the food and feed industry (20–3000 mg/L for Product-Type PT 4) [8] may be ineffective against NTS strains, including those recovered from cases of human infection. This is due to the fact that the MIC/MBC values to PAA for these strains fell within the range of the suggested concentrations to be used in the food and feed industry. The MBC_PAA_ was also found to be higher in the non-MDR subpopulation, which comprised 44% of isolates associated with human infections, thus justifying the overlap of data between sources and antibiotic-resistance profiles.

Significant variations in MICs and MBCs of PAA have been reported among different *Salmonella* strains in various studies [58,59,60,61]. However, these variations, in the ranges of 7–80 mg/L to 500–1760 mg/L for MICs and from 20–80 mg/L to 200–1000 mg/L for MBCs, can be attributed to the diversity of methodological approaches that have been employed. The observed variations can be attributed to factors such as differences in culture medium, incubation temperature, and contact time with the compound as well as the limited number of isolates, serotypes, or clones tested [59,61,62,63], highlighting the need for standardized methods to accurately assess bacteria susceptibility to disinfectants.

## 4. Conclusions

Our study stands out as a unique and comprehensive analysis of the susceptibility of specific populations of NTS and Efm to acidic conditions and PAA, uncovering notable differences among them. Due to its rapid degradation in hydrogen peroxide (which is also unstable) and acetic acid, lack of surface residues, and unspecific mode of action, PAA has been proposed has having a low likelihood of bacterial resistance development [8]. However, while PAA seems to be effective in most disinfection practices, the heightened tolerance observed in NTS associated with human infections and clade A1 or MDR Efm raises concerns and emphasizes the need for ongoing surveillance to monitor the evolution of bacterial tolerance to this environmentally friendly biocide. While acidic pH may play a role in the MDR profile of NTS, it does not seem to have the same impact on Efm, but in both cases, isolates resistant to clinically relevant antibiotics tolerate low pHs values, facilitating animal and human gut colonization. Further research is crucial to investigate the acidic pH and PAA susceptibility of NTS and Efm populations representative of various selective pressure scenarios worldwide, as well as to explore their specific genetic and physiological factors contributing to tolerance to these stresses. As our data indicate that tolerance to PAA and acidic pH appears to be associated with particular populations, the importance of selecting diverse and comprehensive bacterial collections that represent a wide range of scenarios when designing future studies is crucial. Only such an approach will allow drawing robust conclusions not only for NTS and Efm but also for other clinically relevant bacterial genera to which data are still missing [64]. By addressing such research gaps, we can advance our knowledge regarding the survival and persistence of pathogens in food-related and clinical environments, facilitating the implementation of customized control measures and the selection of suitable biocides that are tailored to the local microbiota. Ultimately, these efforts will contribute to more effective strategies in mitigating the impact of diverse antibiotic-resistant pathogens on public health and food safety within a One Health context.

## Data Availability

Data sharing is not applicable to this article.
